# Gaussian-Distributed Codon Frequencies of Genomes

**DOI:** 10.1534/g3.118.200939

**Published:** 2019-02-26

**Authors:** Bohdan B. Khomtchouk, Wolfgang Nonner

**Affiliations:** * Department of Biology, Stanford University, Stanford, CA 94305; † Department of Physiology and Biophysics, University of Miami Miller School of Medicine, Miami, FL 33136

**Keywords:** evolution, genomics, codon usage, genetic code

## Abstract

DNA encodes protein primary structure using 64 different codons to specify 20 different amino acids and a stop signal. Frequencies of codon occurrence when ordered in descending sequence provide a global characterization of a genome’s preference (bias) for using the different codons of the redundant genetic code. Whereas frequency/rank relations have been described by empirical expressions, here we propose a statistical model in which two different forms of codon usage co-exist in a genome. We investigate whether such a model can account for the range of codon usages observed in a large set of genomes from different taxa. The differences in frequency/rank relations across these genomes can be expressed in a single parameter, the proportion of the two codon compartments. One compartment uses different codons with weak bias according to a Gaussian distribution of frequency, the other uses different codons with strong bias. In prokaryotic genomes both compartments appear to be present in a wide range of proportions, whereas in eukaryotic genomes the compartment with Gaussian distribution tends to dominate. Codon frequencies that are Gaussian-distributed suggest that many evolutionary conditions are involved in shaping weakly-biased codon usage, whereas strong bias in codon usage suggests dominance of few evolutionary conditions.

Genome sequencing has provided a large amount of information on how specific genomes use the 64 different codons of the redundant genetic code. This information on codon usage by genomes has raised many questions ranging from the fundamental to the technical: Can codon usage help elucidate the origin of the universal genetic code Crick (1968)? Do genes carry information beyond amino acid sequence in their usage of synonymous codons Zuckerkandl and Pauling (1965)? What are the evolutionary mechanisms and possible (dis)advantages that make genomes use certain different codons more (or less) often than others Zuckerkandl and Pauling (1965); Andersson and Kurland (1990); Duret (2002); Plotkin and Kudla (2011)? How do variations of codon usage arise in evolution Knight *et al.* (2001); Chen *et al.* (2004); Sharp *et al.* (2010); Shah and Gilchrist (2011)? What needs to be understood and controlled to optimize translation in heterologous expression systems Boël *et al.* (2016)?

The detailed information compiled on diversity of codon usages within and across specific genomes has naturally overshadowed a search for common patterns in codon usage that overarch specific codon usages in specific organisms. One such pattern is the descending sequence of genomic codon frequencies. It characterizes bias inherent to the usages of different codons made within a given genome. Intra-genomic bias is necessary for the existence of inter-genomic diversity in the usage of the different codons.

Interest into frequency/rank relations of genomes first concerned linguistic analogies of the ‘language of genes’ and spoken languages Bender and Gill (1986). Empirical formal descriptions from this line of work include a power of rank (Zipf’s law) Bender and Gill (1986); Obst *et al.* (2011), an exponential of rank Tsonis *et al.* (1997); Som *et al.* (2001), and a combination of exponential and linear relations Frappat *et al.* (2003). Codon frequency/rank curves were described by statistical relations with either no external parameter Borodovsky and Gusein-Zade (1989), or two external parameters Naumis and Cocho (2007, 2008). With exception of the statistical model of Borodovsky and Gusein-Zade (1989), these descriptions have not been interpreted with regard to characteristics of evolutionary conditions that shape codon usage. In contrast, to account for observations of inhomogeneous codon usages among genes of the same organism, Gusein-Zade and Borodovsky Gusein-Zade and Borodovsky (1990) have proposed that there exist two compartments with exponentially-distributed codon frequencies.

In this study we investigate intra-genomic bias in codon usage and test the applicability of a generalized two-compartment model in a large set of different genomes. We find that superposition of two compartments of codons with different usages describes genomic frequency/rank plots consistently. Variation of the proportion of the two compartments captures the variations observed among frequency/rank plots of genomes from many taxa. In one compartment, prevailing in eukaryotes, codon usage follows a random pattern as described by a Gaussian distribution of frequencies. Usage bias in that compartment is weak. In the other compartment, usage bias is strong to the extent of using essentially a subset of the available different codons. Prokaryote genomes reveal both compartments in widely varying proportions. The existence of a genomic compartment using codons with Gaussian-distributed frequencies likely implies the existence of many evolutionary conditions, which together could underlie codon usage of eukaryotic genomes.

## Biased Usage Of Codons: Observations

**Figure 1:**
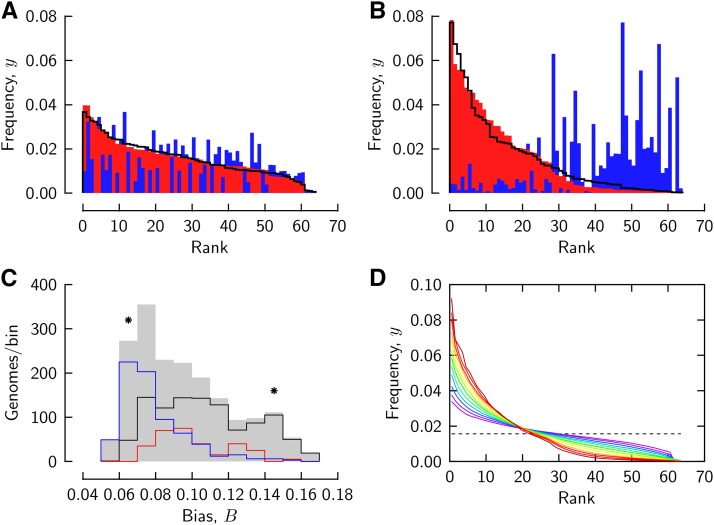
Biased usage of different codons in genomes. Codon frequencies of *Homo sapiens* (A, red color), *Arabidopsis thaliana* (A, blue color), *Streptomyces griseus* (B, red color), and *Clostridium tetani E88* (B, blue color). Frequencies are ordered as described in the text, with the smaller frequency of each two compared genomes shown in the foreground. C: histograms of bias *B* for 1840 genomes from the CUTG database (gray shaded) and of the eubacterial (*black line*), archaeal (*red*, scaled by 5), and eukaryotic subsets (*blue*) of these genomes. The total set comprised 1855 genomes, of which 1840 located to the binned range; the latter are analyzed in this study. Asterisks mark the bins to which the genomes of the panels A,B locate. D: frequency/rank curves averaged over the genomes of each histogram bin in panel C (with bias increasing from purple to red).

Different codons occur with a range of frequencies in a given genome, and any specific codon tends to occur with generally different frequencies across different genomes. These two aspects of bias in codon usage are illustrated in the cases of four organisms sampled from diverse taxa (Figure 1A,B).

In Figure 1A, the frequencies of the 64 different codons in human genomic DNA have been put in descending order (*red color*). When the frequencies of the different codons of the plant *Arabidopsis thaliana* are plotted in the order of the ordered human codons, they do not form a descending sequence (*blue color*). The codon frequencies of the plant, however, when ordered among themselves, form a descending sequence (*line*) that is quite similar to that of the ordered human frequencies. Codon usages of the bacteria *Clostridium tetani* and *Streptomyces griseus* also reveal similar intra-genomic bias, but diverse patterns of inter-genomic bias (Figure 1B). Both forms of bias are stronger in the bacteria than in human and plant. In each of the pairs of genomes of Figure 1A,B, a shared pattern of intra-genomic bias is associated with two distinct patterns of inter-genomic bias.

Here we study the intra-genomic aspect of codon usage bias in the genomic coding DNA of a set of 1840 organisms of different taxa tabulated in the CUTG database Nakamura *et al.* (2000) (files ‘gbxxx.spsum.txt’ where xxx = bct, inv, mam, pln, pri, rod, vrt). We restrict analysis to genomes represented with total codon counts ${\sum^{\text{​}}}_{i=1}^{64}c_{i}\geq 10^{4}$, and define frequencies of occurrence, $y_{i}$, of different codons *i* (1≤i≤64) by:

$$y_{i}=\frac{c_{i}}{{\sum^{\text{​}}}_{i=1}^{64}c_{i}}$$ (1)

For a non-parametric assessment we measure intra-genomic ‘bias’ as:

$$B=\sqrt{\overset{64}{\underset{i=1}{\sum }}{\left(y_{i}-\frac{1}{64}\right)}^{2}}$$ (2)

For a hypothetical genome in which all different codons occur with the uniform frequency 1/64, bias B=0. A real genome will have bias B>0.

A histogram of codon usage bias, *B*, constructed over the entire set of 1840 analyzed genomes is shown in Figure 1C (*gray-shaded*). Histograms drawn as *lines* represent the subsets eubacteria (*black*), archaea (*red*, counts scaled by a factor of 5), and eukaryotes (*blue*). The genomes presented as examples in Figure 1A,B locate to the bins marked by *asterisks*. Weak bias of codon usage is characteristic of eukaryotic genomes, whereas prokaryotic genomes reveal a wide range of bias. Overall, bias varies about threefold over the sampled set of genomes.

Frequency/rank curves averaged over the genomes in each bin of the bias histogram in Figure 1C are shown in Figure 1D. Codon usage bias with respect to average codon frequency (*dashed line*) is observed in all genomes, but a characteristic of the frequency/rank curve changes in the succession of curves from weak (*purple lines*) to strong bias (*red lines*): in curves associated with weak bias, curvature changes direction near the mean frequency, whereas curves associated with strong bias decline without inflection. Moreover, frequency of genomes with strong bias decays in a cascade-like fashion about the lower and upper halves of the ranks.

Frequency/rank relations of a genome assign to a given frequency the number of different codons that have frequencies larger than or equal to that frequency. In this way they define the cumulative distribution of the random variable, codon frequency. For this statistical view of the descending frequency sequence, rotate Figure 1 clockwise by 90 degrees. Then the horizontal axis in panels A,B, and D is the random variable, codon frequency. A position on the vertical axis, read as offset with respect to rank 64, indicates how many different codon values have frequencies less than that at that frequency. The distributions in Figure 1A reveal a sigmoid cumulative distribution, whereas those in Figure 1B resemble an exponential distribution.

In an earlier study of codon usage, Gusein-Zade and Borodovsky Gusein-Zade and Borodovsky (1990) have investigated the possibility that genomes are inhomogeneous due to the existence of gene compartments that make distinct usages of different codons. Specifically, they developed a model of two compartments, each characterized by an exponential distribution. When only one compartment was present in a genome, the distribution was exponential. When both compartments co-existed, the overall frequency distribution was described by the convolution of the two scaled exponential distributions. This model was compared to a small dataset then available to the authors and found to account for characteristics such as the inflection observed in certain frequency/rank relations.

In view of the larger dataset provided by the CUTG database, we noticed that the Gusein-Zade and Borodovsky (GZB) model with two exponentially distributed components falls short regarding the range of bias that it can describe. Here, bias can vary between $1/\sqrt{\left(64\right)}\;=0.125$ and $1/\sqrt{\left(128\right)}\;\approx 0.088$ depending on the proportion of the two compartments. This range is substantially less than the observed bias range (Figure 1C). In this study, we find that a two-compartment model of codon usage describes also the larger dataset of the CUTG database, where variations among genomes are accounted for by a single parameter, the proportion of the two compartments. A generalization of the GZB model, however, is necessary to describe the larger dataset: the distributions that describe codon usage in each compartment need to be chosen differently.

In our generalized model, one compartment uses different codons according to a Gaussian distribution that produces minimal bias. We suppose this compartment to dominate codon usages like those by the genomes in Figure 1A. A Gaussian distribution would be expected to arise due to the Central Limit Theorem of statistics if many different evolutionary conditions shape codon usage in this compartment. Alternatively, the Gaussian compartment could represent many smaller compartments with not necessarily Gaussian distributions of codon frequencies.

The other compartment of our model is governed by a distribution that produces even stronger bias than the exponential distributions proposed by GZB. The exponential distribution, as pointed out by GZB Gusein-Zade and Borodovsky (1990), ‘obeys the principle of maximum diversity of frequencies’, but this does not exclude that more biased distributions are possible (though less likely to arise by chance). For instance, genomes might restrict their machinery of translation to using a subset of different codons within the degenerate genetic code, or even use certain amino acids with preference. Such a codon usage is manifest in the genomes of Figure 1B. In our model, we derive an empirical description of codon frequencies in the second compartment from a subset of the data. We suppose this compartment to dominate in genomes that use different codons with strong bias.

## Construction Of The Model

**Figure 2:**
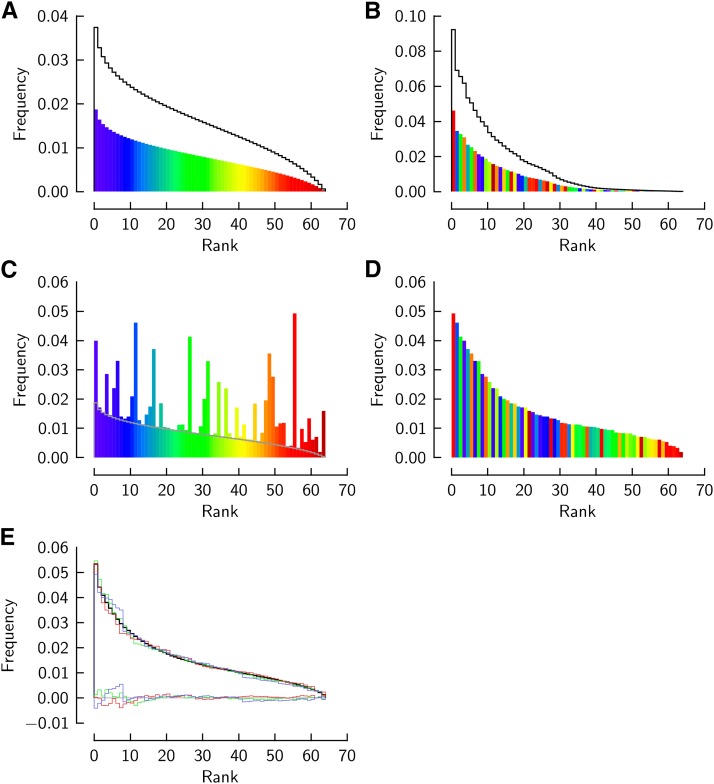
Codon usage in a two-compartment genome model. Frequency sequences computed with Gaussian (A) or empirical frequency distribution (B) (*lines*). *Colored*: frequencies scaled by 1/2 for constructing a joint sequence representing two codon compartments of equal sizes. The colors illustrate different codons associated with the frequency ranks: the order of the second compartment is randomized with regard to the first. (C) Pairwise algebraic sum of the compartmental frequencies of each different codon (matched by color). (D) Frequency sequence (C) after re-ordering for a descending joint sequence. (E) Three samples of frequency sequence like that in (D) computed for different randomizations of the order of different codons in panel B (*colored lines*), and the mean of 100 samples (*black line*). Colored lines along the bottom of the graph: differences between individual samples and the mean.

We describe the model together with a Monte-Carlo numerical approach used to construct it (Figure 2). Our approach reveals (and allows one to assess) a limitation inherent to modeling multiple compartments of codon usage.

Assume a genome comprises two compartments in which, generally, each different codon is used according to two distinct and mutually independent statistical distributions: a Gaussian distribution in compartment 1, and an empirically described distribution in compartment 2. Both distributions are naturally truncated to the range of codon frequency, 0≤y≤1. Overall frequency of a different codon is given by the algebraic sum of the scaled frequencies in the two compartments. If *α* is the proportion of compartment 1,

$$y=\alpha y_{1}+\left(1-\alpha \right)y_{2}$$ (3)

The cumulative probability of frequency $y_{1}$ in compartment 1 is that of a truncated Gaussian distribution,

$$P_{1}\left(y_{1}\right)=\frac{\text{Φ}\left(\frac{y_{1}-\mu }{\sigma }\right)-\text{Φ}\left(\frac{-\mu }{\sigma }\right)}{\text{Φ}\left(\frac{1-\mu }{\sigma }\right)-\text{Φ}\left(\frac{-\mu }{\sigma }\right)}$$ (4)

where Φ denotes the cumulative normal distribution function with position *μ* and scale *σ*. These two parameters are restricted by the requirement that the mean of the truncated distribution be equal to $1/N_{\text{codons}}$ ($N_{\text{codons}}=64$ being the number of different codons). This restriction follows from the normalization inherent to codon frequency. It leads to the relation:

$$1/N_{\text{codons}}=\mu +\frac{\varphi \left(\frac{1-\mu }{\sigma }\right)-\varphi \left(\frac{-\mu }{\sigma }\right)}{\text{Φ}\left(\frac{1-\mu }{\sigma }\right)-\text{Φ}\left(\frac{-\mu }{\sigma }\right)}\times \sigma$$ (5)

where φ(t)=dΦ(t)/dt. Thereby, the Gaussian distribution has only one external parameter. We will choose *σ* and determine *μ* by solving equation 5 for *μ*.

We generate a frequency sequence for compartment 1 using Monte-Carlo sampling of frequency into $N_{\text{codons}}$ discrete bins that are associated with uniform increments of cumulative probability over the range 0<P<1. In each of $10^{6}$ sampling cycles, a cumulative probability value is drawn from a generator of uniform random values in the range 0<R<1. The associated frequency $y_{1}$ is computed by solving equation 4 for $y_{1}$ using a root finder. The frequency $y_{1}$ is included into the frequency average accumulated in the bin associated with the probability interval. A Monte-Carlo sampled frequency sequence is shown in Figure 2A (*solid line*). This calculation needs to be done only once for a chosen Gaussian distribution.

We compute the frequency sequence of compartment 2 empirically, as an average over a group of genomes that reveal the strongest bias of codon usage in our dataset. We average the frequency sequences of the genomes locating to the right-most bin of the bias histogram (Figure 1C). In this study we will not further analyze the statistical basis of this empirical distribution. We will show here that the characteristics of that distribution are universally detected in genomes across taxa, as are the characteristics of a particular Gaussian distribution.

To construct a frequency sequence for a genome comprising, *e.g.*, two equally large codon compartments, we scale the compartmental distribution frequencies by 1/2. We also randomize the association of different codon and frequency rank among the two scaled frequency sequences. This is done by assuming some order of different codons in the ranks of both compartments. Compartment 1 retains that order (represented by a rainbow sequence of colors in Figure 2A), whereas the order is scrambled in compartment 2 by swapping the colors of randomly chosen pairs of ranks in $10^{4}$ cycles (Figure 2B).

We merge the scaled and ordered sequences by algebraically adding to each frequency from compartment 1 the frequency from compartment 2 that is associated with the same different codon (*i.e.*, color). This produces the frequency sequence in Figure 2C, which happens to be no longer in descending order. Re-ordering of that sequence produces the joint frequency sequence of the two codon compartments of the genome (Figure 2D).

The frequency sequence of the compartmentalized genome is intermediate between the compartmental sequences. It also has stochastic roughness that does not exist in the contributing sequences. Both compartmental sequences are thoroughly sampled in the procedure of merging frequencies but each joint frequency is sampled from exactly one pair of frequencies that is formed by a particular different codon in the two compartmental sequences. We cannot expect to model this pseudo-stochastic aspect of joint frequencies because an observed genomic frequency sequence does not reveal how a different codon is associated with rank in each compartment. We can, however, assess the potential consequences of this uncertainty by simulations with the model.

We re-compute the joint frequency sequence using different associations of different codons and frequency ranks in the model compartments (by randomizing the ‘colors’ in compartment 2 for each trial). Figure 2E shows examples of frequency sequence computed in three different trials (*colored lines*) and an average curve computed over 100 trials (*black line*) after re-ordering the sequence from each trial. All individual trials produce frequency sequences that fluctuate with respect to their mean to an extent small enough (rms of $\approx 10^{-3}$) to preserve systematic features of the frequency sequence. These simulations quantify how much the mean theoretical curve, which does not capture consequences of specific ranks of different codons, is expected to diverge from the curve observed in a genome in whose compartments different codons have specific, but unknown ranks.

The computer code for all computations was written from scratch in a PostScript-like language and is available in Zenodo (Khomtchouk and Nonner 2019). Code was executed using a virtual machine (Peyser and Nonner 2011).

### Data availability

The authors affirm that all data necessary for confirming the conclusions of the article are present within the article, figures, source code, and tables.

## Results

We test the generalized two-compartment model on 1840 genomes from the CUTG database. Frequency sequences of the entire genome set are modeled by adjusting only the proportion *α* of the compartment with the Gaussian distribution while maintaining the Gaussian or empirical distributions of each compartment invariant. The scale of the Gaussian distribution is chosen to be σ=0.009, which implies the position μ=0.01461 by equation 5. The standard deviation of the truncated Gaussian distribution is 0.0081, and that of the empirical distribution 0.0207. These compartmental distributions (and α=1/2) have been used in the computations for Figure 2. In optimizing *α* we quantify the closeness of fit by the rms residual between observed and mean theoretical frequencies (the latter determined from 100 trials as described above). This residual is expected to approach $10^{-3}$ for α=0.5 because the theoretical frequency sequence is an average over many different rankings of a different codon’s frequency in the two compartments (Figure 2E).

**Figure 3:**
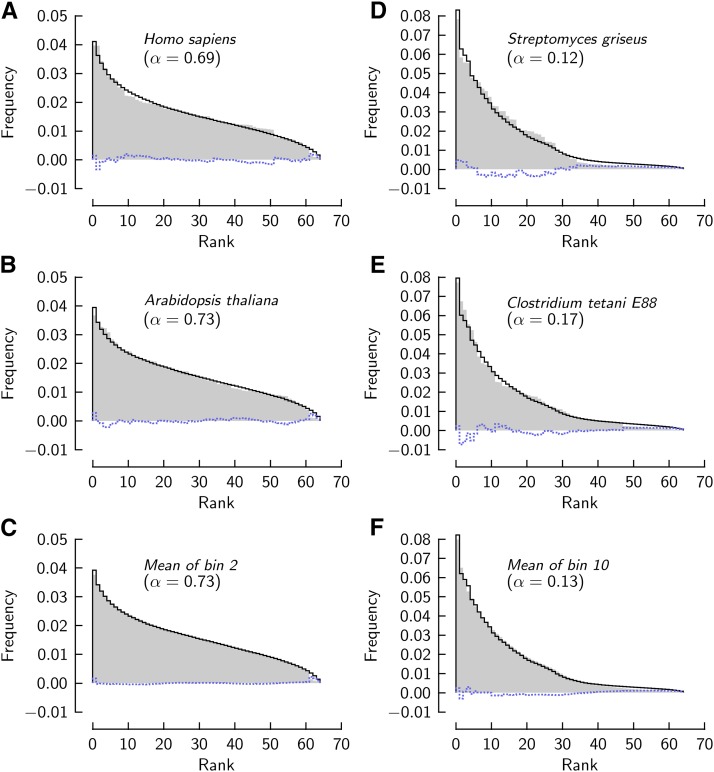
Observed frequencies of codon occurrence compared to theory. *Gray shaded:* ordered sequences of the frequencies of four genomes (A, B, D, E) and ordered frequencies averaged across the subsets of genomes in the two marked histogram bins in Fig. 1C (C, F). Theoretical frequencies (*black lines*) are computed with the indicated proportion, *α*, of the compartment with Gaussian distribution; *dotted lines*: residuals between observed and theoretical frequencies. The rms values of the residuals, scaled by $10^{3}$, are: 1 (A), 0.8 (B), 0.4 (C), 2.2 (D), 1.9 (E), and 1.1 (F).

Figure 3A,B,D,E shows observed and theoretical frequency/rank relations for the genomes introduced in Figure 1A,B. The model describes the data quite well, as is indicated by the residuals of the fit (*dotted lines*).

The specific order of codons in the hypothesized compartments of the individual genomes, which is unknown, might actually limit our model’s capacity to account for the frequencies in an individual genome. On the assumption that different genomes of similar codon usage bias have different specific orders of codons in their compartments (compare Figure 1A,B), we expect that averaging the ordered sequences of frequencies of such genomes produces a frequency sequence that is more accurately predicted by the (averaged) sequence of the model than are sequences of individual genomes. Figure 3C,F compare averaged observed frequency sequence with fitted theoretical sequence. The residuals are substantially smaller than in the case of the individual genomes (Figure 3A,B,D,E; see legend for rms values of residuals). The model describes frequency/rank relations averaged over genomes more accurately than those of individual genomes of similar usage bias.

**Figure 4:**
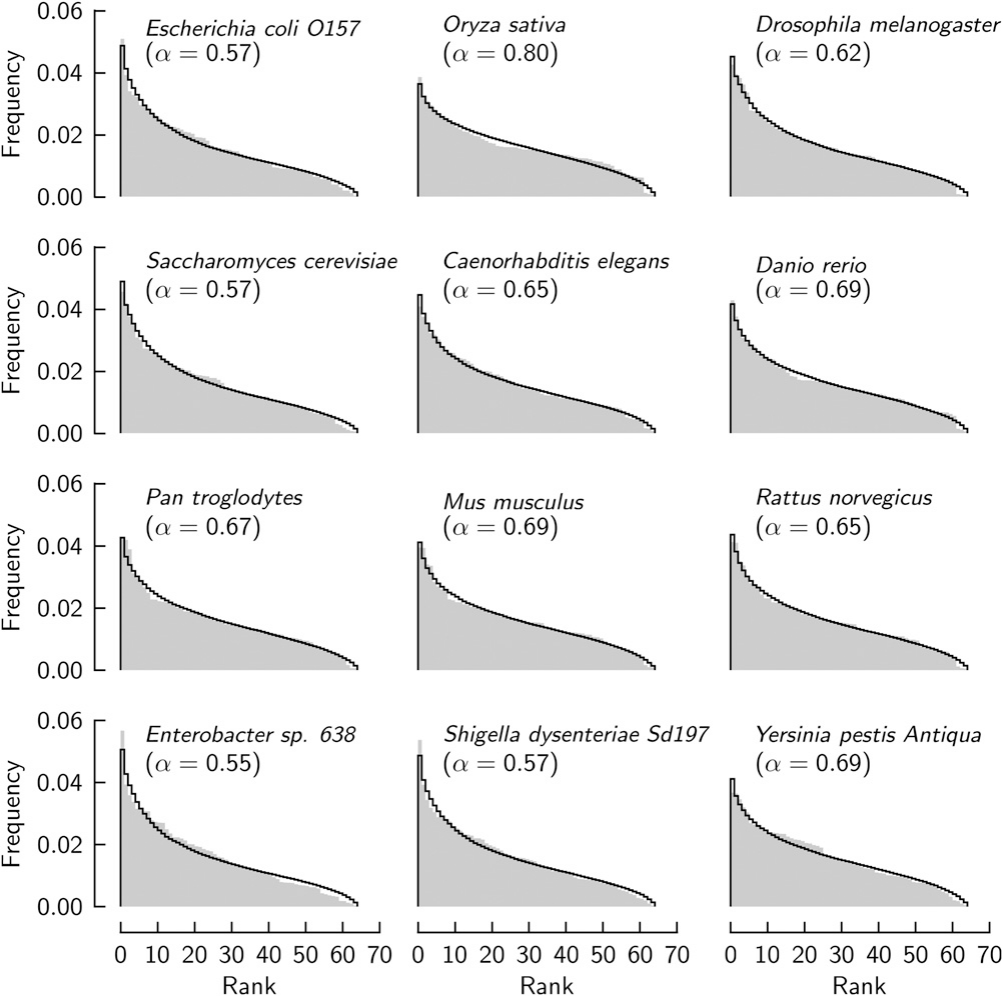
Observed frequencies of codon occurrence compared to theory. Sequences of frequencies of 12 genomes (*gray-shaded*). *Lines* are computed from theory with the indicated proportion, *α*, of the compartment with Gaussian distribution.

Figure 4 extends the comparison of observed and theoretical frequency sequences to twelve genomes that are commonly studied. These genomes belong to diverse taxa, and the bias of their codon usage is in the range most frequently observed in the genomes of the CUTG database. In all cases, adjustment of the proportion of two compartments in the model within the range 0.55≤α≤0.8 allows the model to reproduce these frequency sequences quite well. Although the theoretical frequency sequences are determined by varying substantial contributions from both hypothesized compartments in these cases, the observed sequences are reproduced without any change made to the distributions that are assumed to underlie codon usage in each compartment.

**Figure 5:**
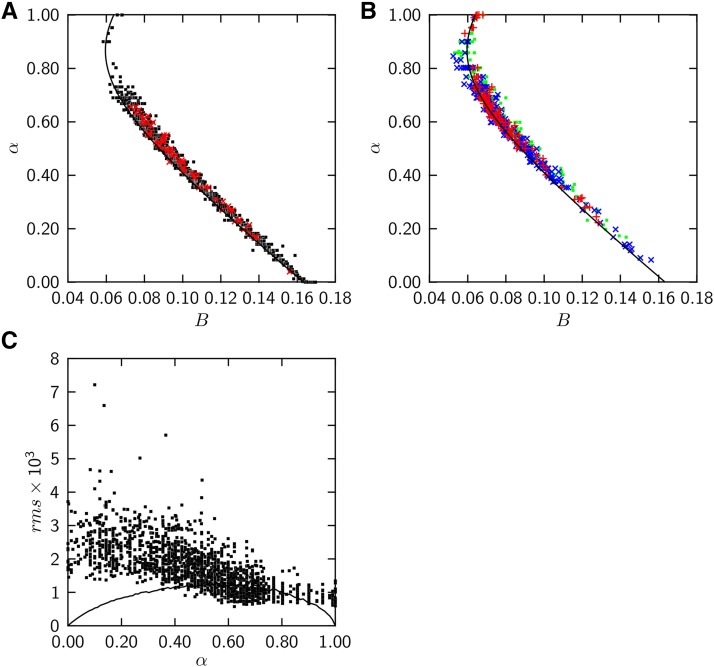
The two-compartment model describes biased codon usage of 1840 genomes. (A) The single varied model parameter *α* describing a genome plotted *vs.* the observed codon usage bias, equation 1: *black symbols* eubacteria, *red symbols* archaea. The *line* represents the theoretical relation, equation 6. (B) Like panel A, representing eukaryotes: *green* plants, *blue* invertebrates, *red* vertebrates. (C) rms of residuals between the observed and theoretical frequency/rank relations (*symbols*) and theoretical rms expected to arise due to unknown ranks of a different codon in the model compartments (*line*).

Figure 5A,B summarize the application of the model to the entire set of genomes, divided between eubacteria and archaea (A) and plants, invertebrates, and vertebrates (B). The proportion of the Gaussian compartment, *α*, which is obtained by the fits of the model to the data, is plotted *vs.* the observed bias (equation 1) for each individual genome (*symbols*). The *lines* show the theoretical relation:

$$B=\sqrt{\left[N_{\text{codons}}-1][\alpha^{2}s_{1}^{2}+{\left(1-\alpha \right)}^{2}s_{2}^{2}\right]}$$ (6)

where $s_{1}=0.0081$ is the standard error of the truncated Gaussian distribution, and $s_{2}=0.0207$ the standard error of the empirical distribution.

Genomes follow the theoretical relation very closely for all taxa represented here, showing that the model produces a consistent relation between compartment proportions and codon usage bias over the full range of bias and of shapes found in the frequency/rank relations of these 1840 genomes.

The accuracy of the model in describing these data are quantified in Figure 5C showing the rms residual between observed and theoretical frequency/rank relations for each genome (*symbols*). The *line* gives the rms residual expected to arise from the uncertain frequency ranks of a different codon in the two model compartments (see Construction of the model and Figure 2E). For the majority of genomes, the actual residual of the fit is similar to the residual expected from the uncertainty of ranks.

## Discussion

We have analyzed aspects of biased usage of different codons at a scale ranging from the individual full genome to many genomes of different taxa. Building on early work of Gusein-Zade and Borodovsky Gusein-Zade and Borodovsky (1990), we have constructed a generalized statistical model to describe the ordered sequence of different-codon frequency in the large genome set of the CUTG database.

Our model posits that codon usage is generally inhomogeneous within a genome, albeit to varying extents. Two compartments of codons provide a good description if each compartment has a distinct distribution of codon frequency. Adjusting a single external parameter, the proportion of the two compartments, while keeping the compartmental frequency distributions the same then suffices to describe codon usage throughout the database.

The two compartmental distributions are ‘antipodal’ in that one is Gaussian with a scale that results in weak bias of codon usage, and the other, described empirically, results in very strong bias. The model is not concerned with a specific map relating the two compartments to the genome’s codons (at whatever scale), nor with a specific association of different codons (or their nucleotide composition) with rank in the ordered frequency sequence. Nevertheless, the model reveals a pattern of codon usages that is apparently universal among genomes.

Existence, within one and the same genome, of two forms of codon usage indicates the existence of ‘inhomogeneous conditions of molecular evolution within a genome’ Gusein-Zade and Borodovsky (1990). Inhomogeneity must be strong enough to drive codon usage into two antipodal compartmental patterns. Of the conditions that support either antipodal distribution of codon frequency, the ones that result in the strongly biased distribution might intuitively appear as the stronger determinants of codon usage. Then, the compartment with Gaussian distribution might be determined more by the absence of strong determinants than by distinguished conditions that favor the more balanced codon usage. Such a simple view of conditions determining codon usage is diffracted by the fact that even the postulated ‘strong’ conditions must generally result in much different ranks of particular different codons in similar ordered sequences of frequency (Figure 1B), a tendency recognizable even in genomes that use codons with less bias (Figure 1A). It is therefore clear that a further discussion of conditions that shape codon usages needs to consider the fate of the specific different codons (a study of this kind will be presented in a separate paper).

In the codon compartment that we model by a Gaussian distribution of frequency, that distribution likely summarizes consequences of many evolutionary conditions as well as outcomes in many subpopulations of the compartment. The Central Limit Theorem of statistics would then imply that the joint distribution approaches a Gaussian, even if individual contributions have non-Gaussian frequency distributions with generally different means and variances. Inhomogeneity of codon usage among genes, or within genes, of a given genome is a common phenomenon, and in many instances has been associated with functional aspects of translation (for recent reviews see Komar (2016); Hanson and Coller (2018)).

The wide range of taxa revealing a Gaussian compartment in genomic codon usage (Figure 5) suggests that conditions contributing to this compartment exist universally across genomes, but does not require that conditions be the same across taxa. On the other hand, our observation that one distribution (of fixed scale and position) describes the Gaussian codon compartment of different genomes suggests the possibility that important evolutionary conditions shaping Gaussian-distributed codon usage are widely shared by genomes.

The strongly biased distribution of the second codon compartment, by virtue of being non-Gaussian, suggests a comparably simple structure informed by few but strong evolutionary conditions. This raises the question of how consequences of both weak and strong evolutionary conditions might co-exist in one genome. An answer might have to include not only the short-term evolutionary status but also the long-term evolutionary history of an organism – the evolutionary conditions informing codon compartments need not co-exist within one and the same span of time.
